# Supplementary material for: Enhanced treatment strategies and distinct disease outcomes among autoantibody-positive and -negative rheumatoid arthritis patients over 25 years: A longitudinal cohort study in the Netherlands
Source: PLoS Med. 2020 Sep 22;17(9):e1003296. doi: 10.1371/journal.pmed.1003296 (PMC7508377; doi:10.1371/journal.pmed.1003296)
Supplement: S1 Fig — (DOCX) [file pmed.1003296.s002.docx]

**S1 Fig:** Flowchart of patient inclusion.

3869 patients presented with early arthritis between 1993-2016 and were included in the Leiden EAC cohort

1377 were RA-patients (clinical diagnosis plus fulfilment of 1987 ACR-criteria)

86 excluded due to randomized clinical trial participation

1285 patients studied

823 Type 1 (autoantibody-positive) RA

462 Type 2 (autoantibody-negative) RA

6 excluded due to unavailable autoantibody-status

**Legend:** Excluded patients (n=92) did not differ from included patients (n=1285) in age, gender and autoantibody-status.
