# Supplementary material for: Enhanced treatment strategies and distinct disease outcomes among autoantibody-positive and -negative rheumatoid arthritis patients over 25 years: A longitudinal cohort study in the Netherlands
Source: PLoS Med. 2020 Sep 22;17(9):e1003296. doi: 10.1371/journal.pmed.1003296 (PMC7508377; doi:10.1371/journal.pmed.1003296)
Supplement: S2 Fig — (DOCX) [file pmed.1003296.s003.docx]

**S2 Fig:** DAG of causal mechanisms to identify potential sources of confounding

Inclusion period

**=**

Proxy treatment strategy

Earlier diagnosis

Earlier start DMARDs

Better first line DMARDs

Treat-to-target treatment adjustments^a^

Suppression disease activity

Shorter symptom duration and less inflammation at diagnosis

Mortality

SDFR

Functionality

Age

Gender^b^

^a^ Increased DAS-scores are promptly followed by treatment adjustments. In case of failure of ≥2 conventional DMARDs biologics are allowed to achieve DAS-remission.

^b^ Since age and gender are related to the outcome but not to the inclusion period, they are formally no confounders.
