# Supplementary material for: Enhanced treatment strategies and distinct disease outcomes among autoantibody-positive and -negative rheumatoid arthritis patients over 25 years: A longitudinal cohort study in the Netherlands
Source: PLoS Med. 2020 Sep 22;17(9):e1003296. doi: 10.1371/journal.pmed.1003296 (PMC7508377; doi:10.1371/journal.pmed.1003296)
Supplement: S3 Fig — (A) Type 1 RA; (B) type 2 RA. (DOCX) [file pmed.1003296.s004.docx]

**S3 Fig:** Percentage of patients achieving DAS28-ESR remission (<2.6) after 1 and 3 years follow-up in type 1 (autoantibody-positive) RA (A) and type 2 (autoantibody-negative) RA (B).

**
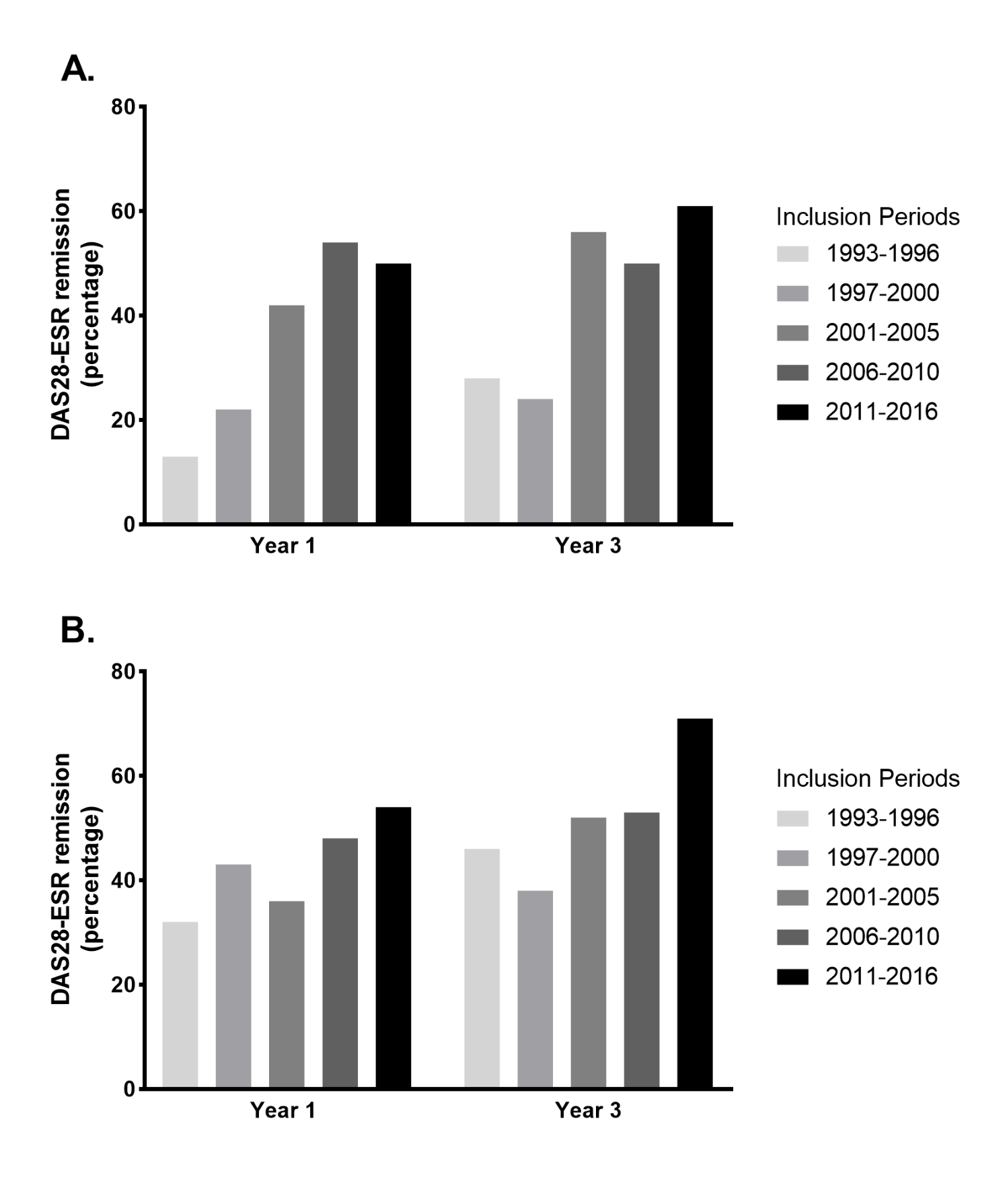
**

**Legend:** P-values for differences between the inclusion periods were <0.01 and <0.01 for type 1 RA and 0.09 and 0.12 for type 2 RA in year 1 and 3, respectively.
