# Supplementary material for: Enhanced treatment strategies and distinct disease outcomes among autoantibody-positive and -negative rheumatoid arthritis patients over 25 years: A longitudinal cohort study in the Netherlands
Source: PLoS Med. 2020 Sep 22;17(9):e1003296. doi: 10.1371/journal.pmed.1003296 (PMC7508377; doi:10.1371/journal.pmed.1003296)
Supplement: S4 Fig — (DOCX) [file pmed.1003296.s005.docx]

**S4 Fig:** Flowchart of RA patients fulfilling the 1987 and/or 2010 criteria

1045 Patients with RA according to both the 1987-RA and 2010-RA criteria

1285 Patients with RA according to the 1987-RA criteria:

64% Type 1; 36% Type 2 RA

1421 Patients with RA according to the 2010-RA criteria

67% Type 1; 33% Type 2 RA

240 Patients with RA according to only the 1987-RA criteria

376 Patients with RA according to only the 2010-RA criteria

3869 Patients presented with early arthritis between

1993-2016

94 Patients excluded due to participation in RCT or missing autoantibodies

92 Patients excluded due to participation in RCT or missing autoantibodies

**Legend:** Patients with RA according to the 2010-criteria partly overlapped with the patients that fulfilled the 1987-criteria, as known from the literature**.** Type 1, autoantibody-positive; type 2, autoantibody-negative.
