# Supplementary material for: Enhanced treatment strategies and distinct disease outcomes among autoantibody-positive and -negative rheumatoid arthritis patients over 25 years: A longitudinal cohort study in the Netherlands
Source: PLoS Med. 2020 Sep 22;17(9):e1003296. doi: 10.1371/journal.pmed.1003296 (PMC7508377; doi:10.1371/journal.pmed.1003296)

**S5 Fig:** Disease activity compared between type 1 (autoantibody-positive) and type 2 (autoantibody-negative) RA


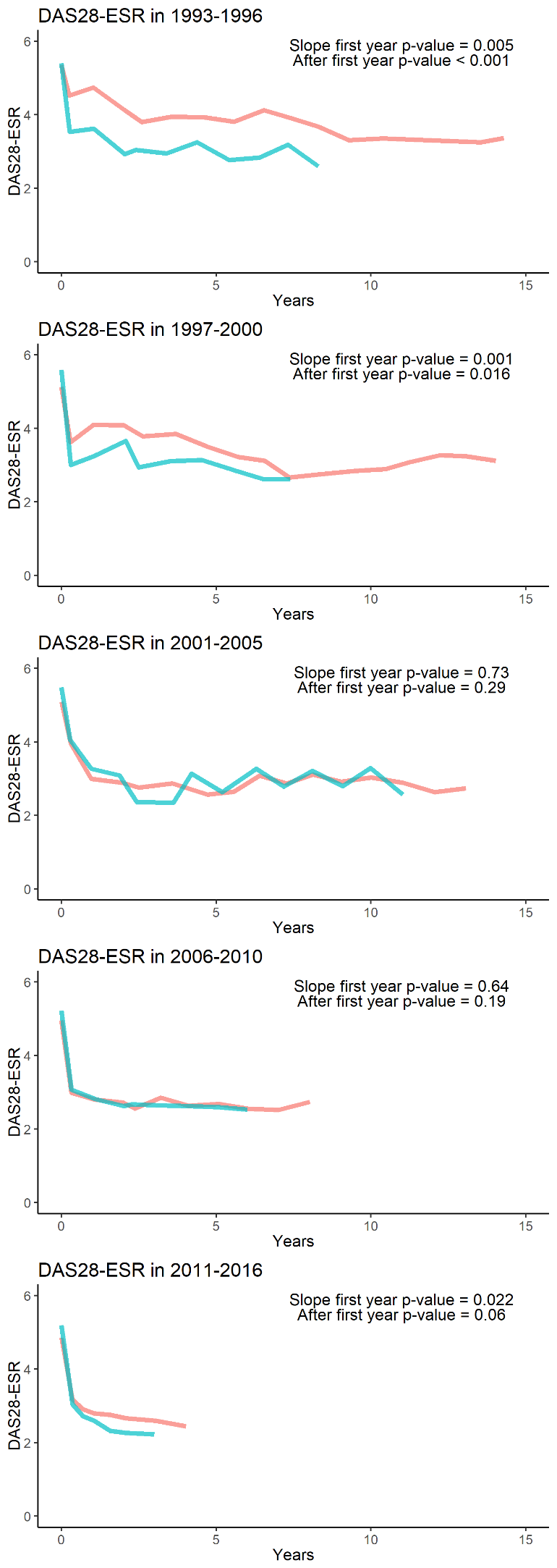

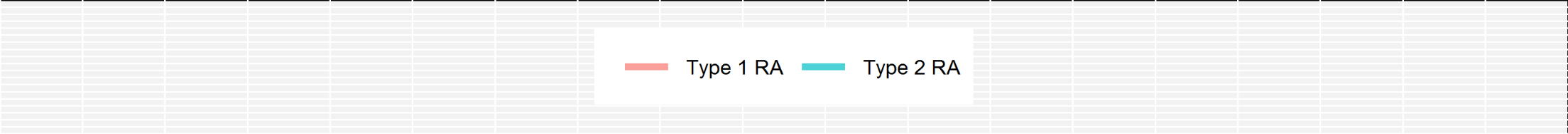

Supplement: S5 Fig — (DOCX) [file pmed.1003296.s006.docx]
