# Supplementary material for: Enhanced treatment strategies and distinct disease outcomes among autoantibody-positive and -negative rheumatoid arthritis patients over 25 years: A longitudinal cohort study in the Netherlands
Source: PLoS Med. 2020 Sep 22;17(9):e1003296. doi: 10.1371/journal.pmed.1003296 (PMC7508377; doi:10.1371/journal.pmed.1003296)

**S9 Fig:** Autoantibody-status over time in RA-patients that were autoantibody-negative at diagnosis, showing that conversion to autoantibody-positivity is rare.

In the figure below, in patients that were autoantibody-negative at baseline, 2% had converted after 1 year and 5% after 2 years. These changes were mostly fluctuations around the cut-off level.


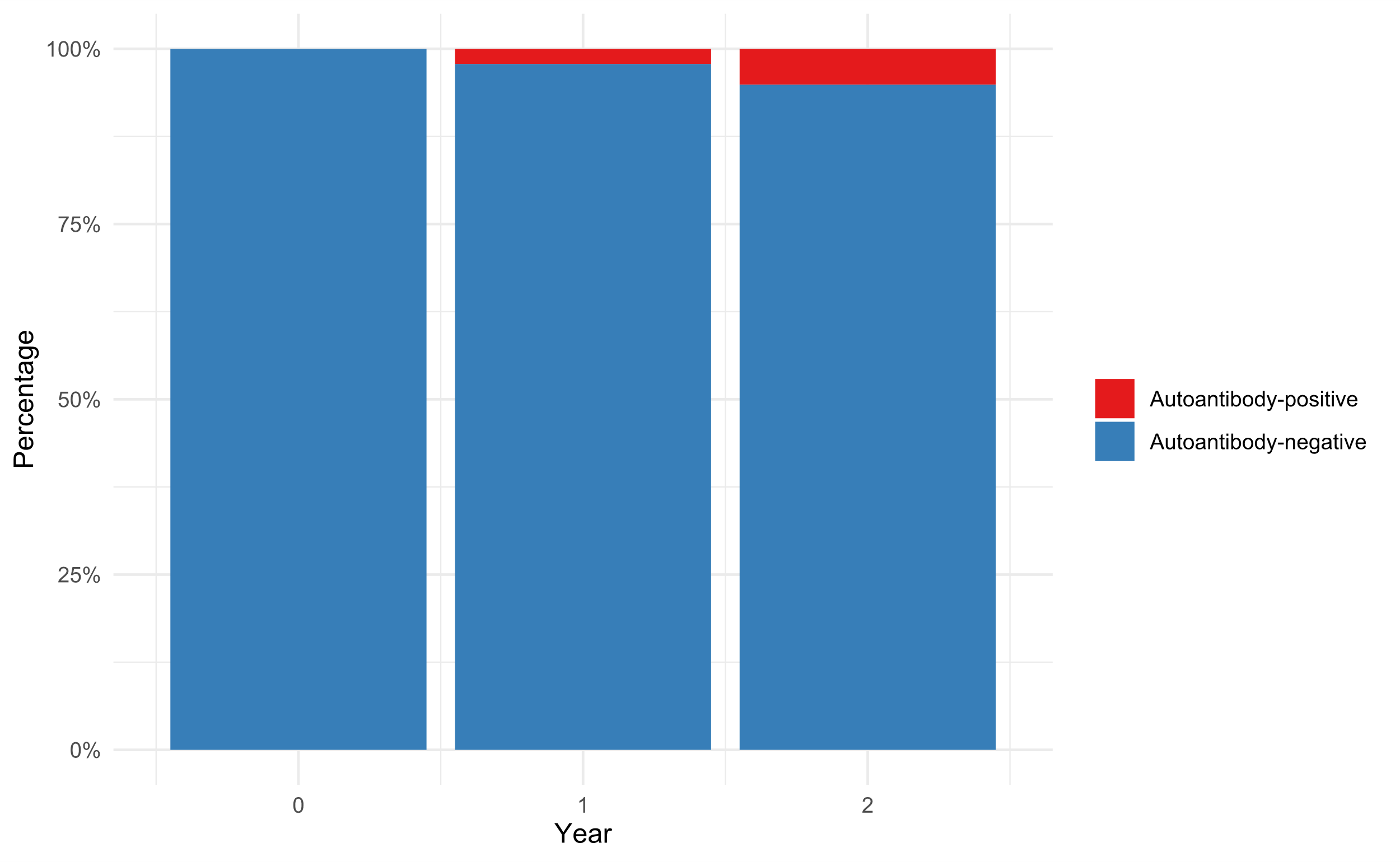

Supplement: S9 Fig — (DOCX) [file pmed.1003296.s010.docx]
