# Supplementary material for: Enhanced treatment strategies and distinct disease outcomes among autoantibody-positive and -negative rheumatoid arthritis patients over 25 years: A longitudinal cohort study in the Netherlands
Source: PLoS Med. 2020 Sep 22;17(9):e1003296. doi: 10.1371/journal.pmed.1003296 (PMC7508377; doi:10.1371/journal.pmed.1003296)
Supplement: S1 Table — (DOCX) [file pmed.1003296.s011.docx]

**S1 Table:** Biologics-use (prevalence within different follow-up durations) per inclusion period, showing slightly more biologics use in type 1 (autoantibody-positive) RA than type 2 (autoantibody-negative) RA.

| **Biologics within 2 years** | **Type 1 RA** | | **Type 2 RA** | |
| --- | --- | --- | --- | --- |
| Inclusion period 1993-1996 | 0.0 | % | 0.0 | % |
| 1997-2000 | 1.0 | % | 0.0 | % |
| 2001-2005 | 4.7 | % | 0.0 | % |
| 2006-2010 | 6.9 | % | 6.8 | % |
| 2011-2016 | 2.2 | % | 0.0 | % |
| **Biologics within 5 years** |  |  |  |  |
| Inclusion period 1993-1996 | 0.0 | % | 0.0 | % |
| 1997-2000 | 5.1 | % | 4.5 | % |
| 2001-2005 | 11.6 | % | 0.0 | % |
| 2006-2010 | 15.3 | % | 9.8 | % |
| 2011-2016 | 11.7 | % | 0.0 | % |
| **Biologics within 10 years** |  |  |  |  |
| Inclusion period 1993-1996 | 5.4 | % | 0.0 | % |
| 1997-2000 | 16.1 | % | 6.0 | % |
| 2001-2005 | 17.8 | % | 2.6 | % |
| 2006-2010 | 18.3 | % | 11.8 | % |
| 2011-2016 | N/A | | N/A | |

**Legend:** Percentages of patients who completed the depicted time period are shown.
