# Supplementary material for: Enhanced treatment strategies and distinct disease outcomes among autoantibody-positive and -negative rheumatoid arthritis patients over 25 years: A longitudinal cohort study in the Netherlands
Source: PLoS Med. 2020 Sep 22;17(9):e1003296. doi: 10.1371/journal.pmed.1003296 (PMC7508377; doi:10.1371/journal.pmed.1003296)
Supplement: S2 Table — (A) Type 1 RA; (B) type 2 RA. (DOCX) [file pmed.1003296.s012.docx]

**S2 Table:** Characteristics of patients with type 1 (autoantibody-positive) (A) and type 2 (autoantibody-negative) RA (B) at first presentation to the EAC that fulfilled the 2010 criteria.

| **A.** | 1993-1996 | | 1997-2000 | | 2001-2005 | | 2006-2010 | | 2011-2016 | |  |
| --- | --- | --- | --- | --- | --- | --- | --- | --- | --- | --- | --- |
|  | (n = 129) | | (n=137) | | (n = 159) | | (n = 250) | | (n = 272) | | p-value |
| Women, n (%) | 86 | (67) | 96 | (70) | 111 | (70) | 167 | (67) | 181 | (67) | 0.92 |
| Age in years, mean (SD) | 55 | (16) | 55 | (16) | 55 | (15) | 55 | (14) | 56 | (15) | 0.69 |
| Symptom duration, days median (IQR) | 143 | (82-292) | 155 | (76-270) | 150 | (80-257) | 150 | (66-274) | 98 | (50-222) | 0.001 |
| Current smoker, n (%) | 39 | (32) | 38 | (30) | 37 | (28) | 49 | (23) | 66 | (26) | 0.37 |
| 28-SJC, median (IQR) | 6 | (3-10) | 6 | (3-12) | 4 | (1-6) | 4 | (1-6) | 3 | (1-6) | <0.001 |
| 28-TJC, median (IQR) | 6 | (2-12) | 6 | (2-14) | 6 | (3-11) | 6 | (3-10) | 4 | (2-9) | 0.001 |
| ESR, median (IQR) | 46 | (26-67) | 33 | (21-54) | 29 | (14-48) | 28 | (14-41) | 28 | (14-43) | <0.001 |
| VAS general health, median (IQR) | 45 | (17-70) | 40 | (25-60) | 51 | (26-70) | 51 | (27-70) | 70 | (50-80) | <0.001 |
| DAS28-ESR, median (IQR) | 5.3 | (4.1-6.3) | 5.1 | (4.1-6.0) | 5.0 | (4.1-5.9) | 4.7 | (3.9-5.8) | 4.7 | (3.8-5.6) | 0.01 |
| HAQ, median (IQR) | 1.0 | (0.5-1.3) | 0.8 | (0.4-1.4) | 0.9 | (0.5-1.4) | 0.9 | (0.5-1.4) | 0.9 | (0.5-1.4) | 0.75 |

|  |  |  |  |  |  |  |  |  |  |  |  |
| --- | --- | --- | --- | --- | --- | --- | --- | --- | --- | --- | --- |
| **B.** | 1993-1996 | | 1997-2000 | | 2001-2005 | | 2006-2010 | | 2011-2016 | |  |
|  | (n = 45) | | (n = 71) | | (n = 88) | | (n = 148) | | (n = 122) | | p-value |
| Women, n (%) | 29 | (64) | 48 | (68) | 63 | (72) | 98 | (66) | 86 | (71) | 0.86 |
| Age in years, mean (SD) | 55 | (15) | 54 | (20) | 60 | (15) | 58 | (17) | 60 | (15) | 0.23 |
| Symptom duration, days median (IQR) | 140 | (78-216) | 126 | (81-260) | 120 | (75-216) | 96 | (54-172) | 76 | (35-213) | 0.02 |
| Current smoker, n (%) | 12 | (27) | 16 | (24) | 18 | (24) | 24 | (19) | 18 | (16) | 0.48 |
| 28-SJC, median (IQR) | 12 | (10-16) | 13 | (8-20) | 6 | (3-10) | 6 | (3-11) | 6 | (2-11) | <0.001 |
| 28-TJC, median (IQR) | 15 | (9-22) | 15 | (8-21) | 12 | (8-20) | 11 | (7-16) | 10 | (6-16) | 0.002 |
| ESR, median (IQR) | 34 | (18-53) | 27 | (14-50) | 26 | (11-44) | 17 | (8-41) | 22 | (9-39) | 0.01 |
| VAS general health, median (IQR) | 39 | (25-64) | 50 | (26-70) | 59 | (38-75) | 64 | (43-80) | 70 | (60-80) | <0.001 |
| DAS28-ESR, median (IQR) | 5.9 | (5.3-6.6) | 5.8 | (4.9-6.5) | 5.6 | (4.5-6.7) | 5.4 | (4.5-6.4) | 5.5 | (4.5-6.3) | 0.23 |
| HAQ, median (IQR) | 1.1 | (0.8-1.6) | 1,0 | (0.6-1.4) | 1.1 | (0.6-1.8) | 1.0 | (0.6-1.7) | 1.0 | (0.6-1.5) | 0.80 |

**Legend:** N, number of patients; SD, standard deviation; IQR, inter quartile range; SJC, swollen joint count; TJC, tender joint count; ESR, erythrocyte sedimentation rate; VAS, visual analogue scale; DAS, disease activity score; HAQ, health assessment questionnaire. p-value; results of Kruskal-Wallis H-test (Fisher’s exact test for proportions and ANOVA for normally distributed variables).

The 28-SJC and 28-TJC counts are the number of swollen and tender joints, respectively, out of 28 joints assessed.

The VAS general health is a self-reported assessment, ranging from 0 to 100.

The DAS28-ESR ranges 2-9.4, with higher scores indicating more disease activity.

The HAQ (HAQ-DI) ranges 0-3, with higher scores indicating more disability.
