# Supplementary material for: Enhanced treatment strategies and distinct disease outcomes among autoantibody-positive and -negative rheumatoid arthritis patients over 25 years: A longitudinal cohort study in the Netherlands
Source: PLoS Med. 2020 Sep 22;17(9):e1003296. doi: 10.1371/journal.pmed.1003296 (PMC7508377; doi:10.1371/journal.pmed.1003296)
Supplement: S3 Table — (DOCX) [file pmed.1003296.s013.docx]

**S3 Table:** Disease activity during the first year and subsequent follow-up and long-term outcomes: sustained DMARD-free remission, mortality and functional disability per inclusion period compared to the reference period for type 1 (autoantibody-positive) RA and fulfilling 2010 criteria.

|  | **DAS28-ESR, slope in first year** | **DAS28-ESR over time after first year** | **Sustained DMARD free remission** | **Mortality** | **HAQ, slope in first year** | **HAQ over time after first year** |
| --- | --- | --- | --- | --- | --- | --- |
|  | Relative mean difference^a^ | Relative mean difference^b^ | Hazard ratio^c^ | Hazard ratio^c^ | Relative mean difference^a^ | Relative mean difference^b^ |
| Inclusion period 1993-1996 | Ref^d^ | Ref^d^ | Ref | Ref | Ref^d^ | Ref^d^ |
| 1997-2000 | -0.13 (-0.96 to 0.70) | 0.04 (-0.42 to 0.51) | 1.00 (0.48 to 2.08) | 0.74 (0.38 to 1.46) | 0.23 (-0.06 to 0.51) | 0.02 (-0.21 to 0.26) |
| 2001-2005 | -0.43 (-1.25 to 0.39) | -0.05 (-0.50 to 0.40) | 1.34 (0.68 to 2.66) | 0.59 (0.29 to 1.17) | 0.21 (-0.12 to 0.54) | 0.14 (-0.09 to 0.36) |
| 2006-2010 | -0.41 (-1.15 to 0.34) | -0.23 (-0.65 to 0.18) | 1.69 (0.86 to 3.31) | 0.80 (0.39 to 1.67) | 0.16 (-0.11 to 0.44) | -0.07 (-0.28 to 0.13) |
| 2011-2016 | -0.72 (-1.46 to 0.02) | **-0.48 (-0.92 to -0.04)** | **2.46 (1.18 to 5.14)** | 0.32 (0.09 to 1.21) | 0.11 (-0.16 to 0.38) | -0.11 (-0.33 to 0.11) |

**Legend:** Bold numbers indicate p-values < 0.05.

^a^ Difference in slope in the first year compared to the slope in 1993-1993 to analyzed with linear mixed models corrected for age and gender. A negative number indicates a steeper slope.

^b^ Difference in mean over time compared the mean over time in 1993-1996 to analyzed with linear mixed models corrected for age and gender.

^c^ Hazard ratios compared to 1993-1996 to analyzed with Cox regression corrected for age and gender.

^d^ The estimated marginal mean, adjusted for age and gender, in type 1 RA for inclusion period 1993-1996 was -0.45 (-0.81 to -0.10) for the slope in DAS28-ESR in the first year, 3.55 (3.37 to 3.73) for DAS28-ESR over time after the first year, -0.17 (-0.31 to -0.04) for slope in HAQ in the first year and 0.74 (0.65 to 0.84) for HAQ over time after the first year.

DAS, disease activity score to ESR, erythrocyte sedimentation rate to HAQ, health assessment questionnaire.
