# Supplementary material for: Enhanced treatment strategies and distinct disease outcomes among autoantibody-positive and -negative rheumatoid arthritis patients over 25 years: A longitudinal cohort study in the Netherlands
Source: PLoS Med. 2020 Sep 22;17(9):e1003296. doi: 10.1371/journal.pmed.1003296 (PMC7508377; doi:10.1371/journal.pmed.1003296)
Supplement: S5 Table — (DOCX) [file pmed.1003296.s015.docx]

**S5 Table:** Disease activity during the first year and subsequent follow-up and long-term outcomes: sustained DMARD-free remission, mortality and functional disability per inclusion period compared to the reference period for type 1 (autoantibody-positive) patients aged <65.

|  | **DAS28-ESR, slope in first year** | **DAS28-ESR over time after first year** | **Sustained DMARD free remission** | **Mortality** | **HAQ, slope in first year** | **HAQ over time, after first year** |
| --- | --- | --- | --- | --- | --- | --- |
|  | Relative mean difference^a^ | Relative mean difference^b^ | Hazard ratio^c^ | Hazard ratio^c^ | Relative mean difference^a^ | Relative mean difference^b^ |
| Inclusion period 1993-1996 | Ref^d^ | Ref^d^ | Ref | Ref | Ref^d^ | Ref^d^ |
| 1997-2000 | -0.44 (-1.01;0.14) | -0.25 (-0.55;0.05) | 0.86 (0.28;2.65) | 1.04 (0.46;2.34) | 0.00 (-0.22;0.23) | 0.04 (-0.10;0.18) |
| 2001-2005 | **-1.73 (-2.33;-1.14)** | **-0.79 (-1.08;-0.49)** | 1.33 (0.48;3.65) | 1.05 (0.48;2.28) | -0.23 (-0.47;0.00) | **-0.15 (-0.29;0.00)** |
| 2006-2010 | **-1.58 (-2.11;-1.05)** | **-0.98 (-1.26;-0.70)** | **2.98 (1.19;7.50)** | 0.78 (0.32;1.88) | **-0.31 (-0.52;-0.10)** | **-0.26 (-0.39;-0.14)** |
| 2011-2016 | **-1.58 (-2.08;-1.08)** | **-0.94 (-1.23;-0.65)** | **4.07 (1.44;11.5)** | 0.57 (0.15;2.21) | **-0.31 (-0.50;-0.12)** | **-0.18 (-0.31;-0.04)** |

Bold numbers indicate p-values < 0.05.

^a^ Difference in slope in the first year compared to the slope in 1993-1993; analyzed with linear mixed models corrected for age and gender. A negative number indicates a steeper slope.

^b^ Difference in mean over time compared the mean over time in 1993-1996; analyzed with linear mixed models corrected for age and gender.

^c^ Hazard ratios compared to 1993-1996; analyzed with Cox regression corrected for age and gender.

^d^ The estimated marginal mean, adjusted for age and gender, in type 1 RA for inclusion period 1993-1996 was -0.20 (-0.64 to 0.24) for the slope in DAS28-ESR in the first year, 3.42 (3.20 to 3.64) for DAS28-ESR over time after the first year, -0.13 (-0.29 to 0.03) for slope in HAQ in the first year and 0.67 (0.57 to 0.78) for HAQ over time after the first year.

DAS, disease activity score; ESR, erythrocyte sedimentation rate; HAQ, health assessment questionnaire.
