# Supplementary material for: Enhanced treatment strategies and distinct disease outcomes among autoantibody-positive and -negative rheumatoid arthritis patients over 25 years: A longitudinal cohort study in the Netherlands
Source: PLoS Med. 2020 Sep 22;17(9):e1003296. doi: 10.1371/journal.pmed.1003296 (PMC7508377; doi:10.1371/journal.pmed.1003296)
Supplement: S6 Table — (DOCX) [file pmed.1003296.s016.docx]

**S6 Table:** Disease activity during the first year and subsequent follow-up and the long-term outcomes: sustained DMARD-free remission, mortality and functional disability per inclusion period compared to the reference period for type 2 (autoantibody-negative) patients aged <65.

|  | **DAS28-ESR, slope in first year** | **DAS28-ESR over time after first year** | **Sustained DMARD free remission** | **Mortality** | **HAQ, slope in first year** | **HAQ over time after first year** |
| --- | --- | --- | --- | --- | --- | --- |
|  | Relative mean difference^a^ | Relative mean difference^b^ | Hazard ratio^c^ | Hazard ratio^c^ | Relative mean difference^a^ | Relative mean difference^b^ |
| Inclusion period 1993-1996 | Ref^d^ | Ref^d^ | Ref | Ref | Ref^d^ | Ref^d^ |
| 1997-2000 | -0.64 (-1.62;0.34) | 0.01 (-0.49;0.50) | 0.44 (0.17;1.13) | 0.87 (0.25;3.04) | 0.09 (-0.28;0.46) | -0.05 (-0.29;0.19) |
| 2001-2005 | -0.63 (-1.59;0.33) | 0.01 (-0.49;0.51) | 1.15 (0.55;2.43) | 0.46 (0.12;1.79) | 0.05 (-0.33;0.43) | 0.13 (-0.11;0.37) |
| 2006-2010 | -0.78 (-1.68;0.12) | -0.30 (-0.77;0.17) | 1.41 (0.69;2.88) | 0.40 (0.10;1.66) | -0.02 (-0.36;0.31) | -0.16 (-0.37;0.05) |
| 2011-2016 | **-0.89 (-1.73;-0.04)** | **-0.53 (-1.05;-0.01)** | 1.89 (0.75;4.78) | 1.13 (0.17;7.61) | -0.12 (-0.43;0.20) | -0.11 (-0.34;0.12) |

Bold numbers indicate p-values < 0.05.

^a^ Difference in slope in the first year compared to the slope in 1993-1993; analyzed with linear mixed models corrected for age and gender. A negative number indicates a steeper slope.

^b^ Difference in mean over time compared the mean over time in 1993-1996; analyzed with linear mixed models corrected for age and gender.

^c^ Hazard ratios compared to 1993-1996; analysed with Cox regression and corrected for age and gender.

^d^ The estimated marginal mean, adjusted for age and gender, in type 2 RA for inclusion period 1993-1996 was -1.10 (-1.77 to -0.43) for the slope in DAS28-ESR in the first year, 2.76 (2.39 to 3.12) for DAS28-ESR over time after the first year, -0.33 (-0.57 to -0.08) for slope in HAQ in the first year and 0.62 (0.45 to 0.79) for HAQ over time after the first year.

DAS, disease activity score; ESR, erythrocyte sedimentation rate; HAQ, health assessment questionnaire.
