# Supplementary material for: Enhanced treatment strategies and distinct disease outcomes among autoantibody-positive and -negative rheumatoid arthritis patients over 25 years: A longitudinal cohort study in the Netherlands
Source: PLoS Med. 2020 Sep 22;17(9):e1003296. doi: 10.1371/journal.pmed.1003296 (PMC7508377; doi:10.1371/journal.pmed.1003296)
Supplement: S7 Table — (DOCX) [file pmed.1003296.s017.docx]

**S7 Table:** Long-term outcomes in type 1 (autoantibody-positive) and type 2 (autoantibody-negative) RA after correction for left-truncation

|  | **Sustained drug free remission** | **Mortality** |
| --- | --- | --- |
| **Type 1 RA** | Hazard ratio^a^ | Hazard ratio^a^ |
| Inclusion period 1993-1996 | Ref | Ref |
| 1997-2000 | 1.21 (0.45 to 3.25) | 0.78 (0.49 to 1.24) |
| 2001-2005 | 1.31 (0.51 to 3.38) | 0.70 (0.45 to 1.11) |
| 2006-2010 | **3.15 (1.36 to 7.30)** | **0.51 (0.30 to 0.87)** |
| 2011-2016 | **4.32 (1.69 to 11.0)** | **0.31 (0.14 to 0.72)** |
| **Type 2 RA** | Hazard ratio^a^ | Hazard ratio^a^ |
| Inclusion period 1993-1996 | Ref | Ref |
| 1997-2000 | 0.60 (0.31 to 1.19) | 0.67 (0.35 to 1.30) |
| 2001-2005 | 0.77 (0.40 to 1.45) | 0.51 (0.25 to 1.03) |
| 2006-2010 | 1.17 (0.64 to 2.10) | 0.67 (0.33 to 1.34) |
| 2011-2016 | **1.98 (1.01 to 3.90)** | 0.34 (0.09 to 1.25) |

**Legend:** Bold numbers indicate p-values < 0.05.

^a^ Hazard ratios compared to 1993-1996, analyzed with Cox regression corrected for age and gender.
