# Supplementary material for: Enhanced treatment strategies and distinct disease outcomes among autoantibody-positive and -negative rheumatoid arthritis patients over 25 years: A longitudinal cohort study in the Netherlands
Source: PLoS Med. 2020 Sep 22;17(9):e1003296. doi: 10.1371/journal.pmed.1003296 (PMC7508377; doi:10.1371/journal.pmed.1003296)
Supplement: S1 Text — (DOCX) [file pmed.1003296.s018.docx]

**S1 Text:** Confounding

The aim of this study was to investigate the influence of the changed treatment strategies on long-term outcomes of type 1 and type 2 RA. To compare treatment strategies, we used inclusion period as a proxy for treatment strategy. Confounders on the association of treatment strategies on long-term outcomes must be associated with the exposure (treatment strategies by proxy of inclusion period), associated with the outcome (disease activity, SDFR, mortality and functional disability) and not be in the causal path.

We did not consider age and gender confounders because they were not associated with the exposure. Nonetheless, we corrected for age and gender to improve model fit. Additional efforts to reduce referral delay were considered as an elemental part of the recent treatment strategies, and therefore we did not regard symptom duration as a confounder because it was considered to be in the causal path.

Reduction of swollen and tender joint counts were considered as a consequence of a reduction of symptom duration and consequently part of the causal path. A visual summary of these considerations can be found in the DAG in the figure on the next page.
