# Supplementary material for: Enhanced treatment strategies and distinct disease outcomes among autoantibody-positive and -negative rheumatoid arthritis patients over 25 years: A longitudinal cohort study in the Netherlands
Source: PLoS Med. 2020 Sep 22;17(9):e1003296. doi: 10.1371/journal.pmed.1003296 (PMC7508377; doi:10.1371/journal.pmed.1003296)
Supplement: S2 Text — (DOCX) [file pmed.1003296.s019.docx]

**S2 Text:** Additional R-packages used

Cowplot 1.0.0

Emmeans 1.4.2

Extrafont 0.17

Ggfortify 0.4.7

Haven 2.1.1

Here 0.1

Hmisc 4.2-0

Viridis 0.5.1

Knitr 1.25

Lme4 1.1-21

Mice 3.6.0

Nlme 3.1-140

Pander 0.6.3

Readr 1.3.1

Rms 5.1-3.1

Skimr 1.0.7

Survival 2.44-1.1

Survminer 0.4.6

Tidyverse 1.2.1
