# Supplementary material for: Enhanced treatment strategies and distinct disease outcomes among autoantibody-positive and -negative rheumatoid arthritis patients over 25 years: A longitudinal cohort study in the Netherlands
Source: PLoS Med. 2020 Sep 22;17(9):e1003296. doi: 10.1371/journal.pmed.1003296 (PMC7508377; doi:10.1371/journal.pmed.1003296)
Supplement: Attachment — Submitted filename: Point by point FINAL.docx [file pmed.1003296.s020.docx]

**Requests from the editors:**

***In responding to the reviewers' points, the issue raised by the statistical reviewer (that the analysis currently does not include a between-group comparison but only within-groups) should be fully dealt with, as this seems crucial.**

Answer: We thank the reviewers and the editor for this suggestion. We fully agree that comparison of change between groups is an interesting addition that will enable to quantify how much improvements differs between both disease types. Therefore we added a new analysis to the main body of the article. In this analysis, we compared the disease outcomes between the two types of RA and found results in line with the main message of the article, namely more improvement in long-term outcomes in autoantibody-positive RA. Methods, results and discussion of results can be found in the main paper on page 11, 15-16, 19, Table 4. We agree with the reviewers and the editor that these analyses improved the quality of the article.

***Please revise your title according to PLOS Medicine's style. Your title must be nondeclarative (ie not state the findings), rather beginning with the main concept if possible. Please place the study design in the subtitle (ie, after a colon) - eg in this case "prospective cohort".**

Answer: Thank you. We adjusted the title accordingly to: *“Differences in improvement of disease outcomes between autoantibody-positive and autoantibody-negative rheumatoid arthritis with enhanced treatment strategies over 25 years: A longitudinal prospective cohort study”*

***In the last sentence of the Abstract Methods and Findings section, please describe the main limitation(s) of the study's methodology.**

Answer: We thank the editor for this suggestion. We added the main limitation that was also mentioned by the reviewers, namely the use of instrumental variables for treatment strategy, to the abstract on page 3.

***At this stage, we ask that you include a short, non-technical Author Summary of your research to make findings accessible to a wide audience that includes both scientists and non-scientists. The Author Summary should immediately follow the Abstract in your revised manuscript. This text is subject to editorial change and should be distinct from the scientific abstract. Please see our author guidelines for more information:** [**https://journals.plos.org/plosmedicine/s/revising-your-manuscript#loc-author-summary**](https://journals.plos.org/plosmedicine/s/revising-your-manuscript#loc-author-summary)

Answer: Thank you. As suggested, we added an Author Summary on page 4-5.

***In the methods section, where you currently state that local ethical approval was obtained, would suggest also naming the ethics board/committee that gave approval (this is stated in some of the additional information with the paper, but good if it can be updated in the paper itself as well).**

Answer: We agree and added the name of our ethics committee on page 8.

***Please present and organize the Discussion as follows: a short, clear summary of the article's findings; what the study adds to existing research and where and why the results may differ from previous research; strengths and limitations of the study; implications and next steps for research, clinical practice, and/or public policy; one-paragraph conclusion.**

Answer: We apologize for this unclarity. We structured the discussion accordingly on page 16-20.

***When you cover limitations in the Discussion, it would be good to say something about the intended purpose of the instrumental variable analysis in this study. How well does the use of time period fulfill the assumptions of such an analysis? How well does this approach begin to deal with some of the limitations of a non-instrumented-for analysis?**

Answer: Thank you for pointing out that we could emphasize more on the strong points and assumptions of instrumental variable analysis. We added a paragraph to the discussion in which we elaborate on this on page 18.

***Please ensure that the study is reported according to the STROBE guideline, and include the completed STROBE checklist as Supporting Information. 1 Please add the following statement, or similar, to the Methods: "****This study is reported as per the Strengthening the Reporting of Observational Studies in Epidemiology (STROBE) guideline (SChecklist)." The STROBE guideline can be found here: http://www.equator-network.org/reporting-guidelines/strobe/. When completing the checklist, please use section and paragraph numbers, rather than page numbers.**

Answer: Thank you. We completed and added a STROBE checklist in the supplementary files and the corresponding statement in the methods on page 13.

***Did your study have a prospective protocol or analysis plan? Please state this (either way) early in the Methods section.**

**a) If a prospective analysis plan (from your funding proposal, IRB or other ethics committee submission, study protocol, or other planning document written before analyzing the data) was used in designing the study, please include the relevant prospectively written document with your revised manuscript as a Supporting Information file to be published alongside your study, and cite it in the Methods section. A legend for this file should be included at the end of your manuscript.**

**b) If no such document exists, please make sure that the Methods section transparently describes when analyses were planned, and when/why any data-driven changes to analyses took place.**

**c) In either case, changes in the analysis-- including those made in response to peer review comments-- should be identified as such in the Methods section of the paper, with rationale.**

Answer: Unfortunately a formal prospective analysis plan was not written down and submitted prior to performing the analyses. The analyses were performed in response to an invitation of Dr. B. Jopke in 02/2018 in which he informed whether we had long-term outcome data available on the improved prognosis of RA, which could be studied. Because our 25-year old cohort is indeed suitable to study this, we performed the analyses following this invitation but have not submitted a formal analysis plan. We added a description of when the analysis was planned on page 7, 11 and 13.

*-----------------------------------------------------------*

**Comments from the reviewers:**

**Reviewer #1: Alex McConnachie, Statistical Review**

**Matthijssen et al present an analysis of data from a large cohort of RA patients. They use period of entry to the cohort as a proxy for the type of treatment received, and analyse four long term outcomes (disease activity, remission, survival, and disability). This review considers the statistical aspects of the paper.**

**The use of time period as an instrumental variable is acceptable, even though the authors subsume earlier diagnosis within improved treatment protocols. The methods of analysis used for each outcome are also good.**

Answer: We thank the reviewer for this positive comment.

**For me, the main problem with the paper is the lack of comparison between the two disease subtypes. Results are generally presented for each outcome, within each subtype, but do not compare the two types directly. The authors conclude, based on statistically significant results within one group but not the other, that the two groups are different. This is not enough; the results should be presented in such a way as to test whether the two groups are different. There is no statistical evidence presented that supports the main conclusion of the paper.**

Answer: We fully agree with this comment that was also raised by the editor. Please also see our answer above. We added analyses that compared the two types directly in the main body of the manuscript (page 11, 15-16, 19, Table 4). Most importantly, these analyses agreed with the main message of the article, as we observed a difference between the two groups as a greater improvement in long-term outcomes in autoantibody positive RA was observed. We thank the reviewer for this suggestion, which clearly improved the article.

**In the abstract, it is not clear why two HRs are presented for SDFR rates in type 1 RA. Only when looking at the tables is it obvious what these HRs represent. Also, when reporting HAQ improvements, it is not clear what is being reported. Also, the confidence limits are the wrong way round (negative changes are being reported as improvements, by removing the minus signs).**

Answer: We thank the reviewer for pointing out this unclarity. We further clarified this on page 2-3.

*-----------------------------------------------------------*

**Reviewer #2:**

**Interesting but on review of this revision I think the designation of Type 1 and Type 2 is not quite right. Really this is just ACPA and/or RF positive vs. ACPA and/or RF negative RA and without trying to bring in other autoantibodies, even if not standard in RA diagnostic yet (e.g. anti-CarP, ANAs, other), or features of disease (nodules etc) it seems too much of a stretch to label these subsets Type 1 and 2 and trying to make a case for any nomenclature change. I think still very interesting but perhaps revise to just state that this is analyses of ACPA and/or RF positive vs. negative, and then in the discussion section discuss how this may for the basis for future classification.**

Answer: We thank the reviewer for this positive comment about our manuscript. We fully agree with the reviewer that divisions between diseases should be made with caution and only after careful consideration. However, it should also be avoided to make suggestions on such distinctions too late, as this may hamper optimal research and resulting treatment of RA patients. In daily practice, the distinction between autoantibody-negative and autoantibody-positive disease is already frequently used and many research articles on different aspects of RA confirm this distinction.

However we see the point of the reviewer and made several changes in response to this comment to reduce the emphasis on the two types:

- In the abstract we removed the words ‘type 1 and type 2” from the introduction, aim, methods and results and only included a suggestion in the last line of the abstract.
- It was also removed from the introduction section of the manuscript.
- In the manuscript, method section, we describe the choices that we made in subdividing patients. This section is now expanded and we have included the notion that future studies may reveal that variables other than ACPA and RF may be of additional importance to optimize the distinction of relevant groups with differences in pathogenesis and response to treatment. Please see page 9.
- We now also elaborated on this issue in the discussion, please see page 17.

**Literature supports that subjects who are seroneg at initial diagnosis may later become positive. was this evaluated?**

Answer: We thank the reviewer for this suggestion. We agree that conversion of auto-antibodies is a very interesting topic. A paper on seroconversion from autoantibody-positivity to -negativity in the Leiden EAC cohort has already been published (Boeters et al. ARD 2019). With regard to conversion from autoantibody negativity o autoantibody-positivity: this has been studied in the past in data from our own cohort, but this was not published. Please see the figure below; 2% had converted after 1 year and 5% after 2 years. These changes were mostly fluctuations around the cut-off level.

Our data showing the infrequence of seroconversion, in both directions, has resulted in the decision on the past to stop the yearly measurements of auto-antibody status. We have now added information on this issue to the discussion section please see page 17-18. (We incorporated the numbers in text, but the figure presented below was not added. If the reviewer insists we are willing to add this figure to the supplementary materials).

Autoantibody-status over time in patients that were autoantibody-negative at diagnosis, showing that conversion to autoantibody-positivity is rare.

**please list the specific ACPA and RF assays used and also comment on how seropositivity may differ based on the test used**

Answer: We thank the reviewer for this excellent suggestion and the requested information is included in the main article on page 8. Moreover, the influence of the cut-off is now discussed on page 17-18.

**Please provide analyses of how various elements of disease activity measures may differ between disease subsets. for example, is CRP or swollen joints comprising more of the score in one group vs. global scores in another**

Answer: Thank you. We agree with the reviewer that the distribution of the various elements of disease activity are of great interest. From Table 1 it can be concluded that autoantibody-negative RA presented at the time of diagnosis with slightly higher disease activity scores and also slightly higher swollen and tender joint counts.

In response to this comment we have now also determined the attribution of the components to the DAS28 score. Results can be found in the table below. No large differences between the two subsets can be observed; It is therefore expected that differences in attribution of DAS components between the disease subsets do not influence our results.

Because we did not see important differences in the attribution of the different components to the DAS scores between the groups, we have not included this table in the manuscript itself. However If the reviewer insists that this table should be included, we are certainly willing to accommodate this and add this table to the supplementary materials.

| Percentage attribution of the DAS components at baseline | | | | |
| --- | --- | --- | --- | --- |
| **Autoantibody-positive RA** | 28-SJC | 28-TJC | ESR | VAS general health |
| 1993-1996 | 13% | 26% | 50% | 11% |
| 1997-2000 | 14% | 27% | 47% | 13% |
| 2001-2005 | 11% | 28% | 47% | 14% |
| 2006-2010 | 11% | 28% | 46% | 15% |
| 2011-2016 | 11% | 24% | 46% | 18% |
| **Autoantibody-negative RA** |  |  |  |  |
| 1993-1996 | 14% | 27% | 47% | 12% |
| 1997-2000 | 16% | 32% | 40% | 11% |
| 2001-2005 | 12% | 33% | 41% | 14% |
| 2006-2010 | 12% | 30% | 42% | 16% |
| 2011-2016 | 13% | 27% | 41% | 19% |

**please discuss how diagnostic certainty may influence the use of biologics - this can be separate issue from disease severity. for example, a practitioner may be more willing to use expensive and perhaps toxic meds for seropositive RA because they are sure of their diagnosis based on the blood test.**

Answer: We thank the reviewer for this comment. We believe that autoantibody-status was of limited importance to prescribe biologics in our patients. The rules of prescribing biologics have not changed in the last two decennia in our country and center. Biologics are prescribed in patients that failed on ≥2 conventional DMARDs. To prevent differences between rheumatologists, all patients starting biologics are discussed in a weekly meeting, where decisions are based on disease activity and its consequences such as functional limitations and not on autoantibody-status. Furthermore all patients in our study had a clinical diagnosis of RA and fulfilled the 1987 classification criteria of RA.

However, because of the known differences between the classification criteria, analyses were repeated in patients with a clinical diagnosis of RA that fulfilled the 2010 criteria for RA. Similar results were obtained, please see Supplementary Table 2-4.

**1987 criteria don't include ACPA - how was this handled in RF negative, ACPA positive subjects? could they have been excludedbecause they may not have met criteria?**

Answer: Thank you. We understand the concern about exclusion of RF negative, ACPA positive patients. However, inclusion of the EAC cohort is based only on the presence of early arthritis. Fulfillment of the criteria and partly also ACPA-positivity were assessed retrospectively (in the patients included in the ‘90s stored serum samples were used because the ACPA test became available after 2006). Because of unchanged inclusion criteria and the retrospective selection of patients, we could select similar patients from all time periods.

Furthermore, as described above, in sensitivity analyses the 2010 criteria were used instead of the 1987 criteria. Similar results were obtained.

*-----------------------------------------------------------*

**Reviewer #3:**

**Matthijssen and co-authors report here on the long term outcomes of autoantibody-positive and -negative rheumatoid arthritis (RA) over 15 years of follow-up. Outcomes of interest are drug-free remission, functional disability and mortality. Data were retrieved from the large Leiden Early Arthritis inception cohort. All analyses were adjusted for the inclusion period, taking into account important modifications that have occurred in the management of RA over the years (earlier diagnosis, use of methotrexate as first DMARD, treat-to-target strategies). Collectively, the Authors show that all the outcomes analyzed ameliorated over time in autoantibody-positive but not autoantibody-negative RA.**

**Data arising from the current research work are of interest as they open the interesting perspective on whether autoantibody-positive and -negative RA should be managed as two different diseases entities.**

Answer: We thank the reviewer for this positive comment.

**However, a number of confounders should be carefully evaluated before drawing definitive conclusions.**

**Major points to consider:**

**1) All the outcomes analyzed are strongly dependent of the treatment strategy adopted. Although the Authors show similar trends in the reduction of disease activity in autoantibody-positive and -negative patients, it cannot be excluded that differences in the observed outcomes may depend on different treatment strategies. Patients with autoantibody-negative RA may indeed receive less frequent combination therapy with biological drugs. This appears clearly evident from Table S1. Also the use of glucocorticoids may differ. It is therefore important that the Authors provide information on (and correct their analyses for):**

**- the trends in drug-free remission, functional disability and mortality in patients treated with csDMARDs and in those also receiving bDMARDs stratified for the autoantibody status. Indeed, I believe that adjusting analyses for inclusion period may not take into account the use of bDMARDs. From Table S1, it appears that bDMARDs have increased in autoantibody-positive but not in -negative RA in more recent times.**

**- the use of glucocorticoids in autoantibody-positive and -negative patients**

Answer: We agree with the reviewer that precise medication data could deepen our understanding of how the difference in change between the two disease types came to be and which type might benefit more from certain therapies. However, using individual treatment data without randomization could introduce confounding by indication as only patients with very severe disease are prescribed biologics.

Therefore, instead of individual treatment data, we used inclusion period as instrumental variable for treatment strategy. As an alternative to randomization, instrumental variable analysis uses a proxy (inclusion period) to create groups with comparable patients that receive different treatment strategies. Between these groups, treatment strategies can be compared without confounding by indication, under the assumption that allocation to the groups is random. Since inclusion criteria of the Leiden EAC have not changed over time, year of RA diagnosis was assumed random. Correcting for the treatments that were received could reintroduce confounding by indication. We added a discussion on this on page 18-19.

**2) As the Authors acknowledge, autoantibody-negative RA may itself be a heterogeneous disease entity. Among the various confounders, elderly-onset RA has peculiar clinical features in presentation and outcomes. From Table 1, it appears autoantibody-negative patients are (as expected) older. I would therefore suggest that the Authors perform a subanalysis on patients (both autoantibody-positive and -negative) aged less than 65 years. I am aware that this could limit the frequency of observed events for the outcome of mortality, but other outcomes remain valid.**

Answer: We thank the reviewer for this suggestion. It is true that autoantibody-negative RA patients are more frequently older and it is interesting to see how this might influence our results. As suggested, we added a sensitivity analysis in the main paper on page 13 and in Supplementary Table 5&6. We obtained similar results in this subgroup except for the long term outcome mortality. These differing results for mortality might be caused by a lower number of events, as the reviewer already foresaw. This is also discussed on page 19.

We have phrased in the method section that this sensitivity analysis was performed on suggestion of the reviewer, as to our understanding this is in line with the journal guidelines. However, if we have misunderstood the guidelines, we are certainly willing to rephrase this.

**Additional points:**

**The scope of the current study was a within-subgroup comparison of outcomes over time, and not a direct comparison between autoantibody-positive and -negative RA.**

**However, from the Kaplain Maier curves presented, it seems that mortality and functional disability have remained poorer in autoantibody-negative RA compared to autoantibody-positive even after the adoption of T2T.**

Answer: We fully agree with this comment, thank you. The editor and Reviewer #1 also raised this point. Therefore we added a direct comparison to the main body of the article (page 11, 15-16, 19, Table 4). Most importantly, this comparison was in line with the main message of the article, namely more improvement in long-term outcomes in autoantibody-positive RA. We thank the reviewers and the editor for this suggestion as it clearly improved the article.

**To avoid misinterpretations, it would be important to provide the reader with numbers of in the Kaplan Meier curves. Is the percentage of losts to follow-up different between autoantibody-positive and -negative RA? This would help understanding whether the current analyses have selected particular patient populations.**

Answer: We agree with the reviewer that drop-out is of vital importance to consider when analyzing a long-lasting prospective cohort. The numbers belonging to the Kaplan Meier curves are now added and there were no relevant differences in drop-out between the two groups

Mortality was extracted from the civic registries at a specific time, all “drop-outs” were caused by follow-up reaching current time. Therefore, no differences between autoantibody-positive RA and autoantibody-negative RA or the inclusion periods can exist. Indeed percentages at risk for all inclusion periods were similar for the two groups.

For SDFR, numbers at risk are also showing in the legend of Figure 1&2. Please bear in mind that the presented percentage at risk are not only the result of drop-out but also to achieving SDFR. SDFR is much more often achieved in autoantibody-negative RA and this causes the difference in percentage of ‘patients at risk’. In other words, a higher percentage of SDFR at one time point can therefore result in a lower percentage of patients being “at risk” without additional drop-out. For example, 85% of inclusion cohort 1993-1996 of autoantibody-positive RA are still at risk for SDFR at 5 years, compared to 73% of autoantibody-negative RA. However, only 2% of autoantibody-positive RA reached SDFR at that time point, compared to 14% in autoantibody-negative RA. Therefore the percentage lost to follow-up is similar for both types: 13% in autoantibody-positive RA, compared to 14% in autoantibody-negative RA. From the Kaplan Meijer curves it can be concluded that this also holds for the other inclusion periods and other time points.

Concludingly, because drop-out is similar between types and inclusion periods for both outcomes, we consider the influence of lost-to follow-up to be minimal in the analysis of the Kaplan Meijer curves.
