# Supplementary material for: Enhanced treatment strategies and distinct disease outcomes among autoantibody-positive and -negative rheumatoid arthritis patients over 25 years: A longitudinal cohort study in the Netherlands
Source: PLoS Med. 2020 Sep 22;17(9):e1003296. doi: 10.1371/journal.pmed.1003296 (PMC7508377; doi:10.1371/journal.pmed.1003296)
Supplement: Attachment — Submitted filename: point by point part2.docx [file pmed.1003296.s021.docx]

**PART 2**

**EDITORIAL REQUIREMENTS:**

**1. Please double check the STROBE checklist – items after 15 seem to have been cut off.**

Answer: Thank you for pointing out this error, we have corrected the STROBE checklist.

**2.	Data Availability Statement (DAS): Please include an appropriate contact (web or email address) for inquiries, eg. the ethics committee that approved the protocol; this contact cannot be a study author. You may have removed this during the most recent revision.**

Answer: Thank you, we have added an email address to the DAS.

**3.	Abstract: Please quantify the following with 95% CIs and p values: “Similarly, functional disability … but not in autoantibody-negative RA” (p. 3, line 11); as you have done for the other part of the sentence.**

Answer: Thank you, we have added the confidence intervals on page 3, line 11-12.

**4.	Author Summary: Please correct typo: “distict” (p. 4, line 7).**

Answer: Thank you, we have corrected the type on page 4, line 7.

**PRODUCTION REQUIREMENTS:**

**1. Please ensure that all author names are spelled correctly in the manuscript file and the submission form, including the use of a middle name/initials, and any special characters (accents, umlauts, etc.). Please place first/given names before last/family names. We ask that you ensure they match verbatim. Please be aware that correct spelling and formatting of author names in the article file is vital for accurate indexing in PubMed.**

Answer: Thank you, we have checked and confirmed that the author names are spelled correctly.

**2. Please ensure that each supporting information file has a legend at the end of the manuscript file, after the Reference list.**

Answer: Thank you, we have added the legends of the supporting information files after the reference list.

**3. Please note that your submission must refer to specific files using our naming convention (S1 Fig, S1 Table, S1 Text, S1 Data, etc.) Please update all in-text citations and supporting information legends/captions accordingly.**

Answer: Thank you, we have corrected the captions accordingly.

**PART 3:**

**1. Please ensure that all author names are spelled correctly in the manuscript file and the submission form, including the use of a middle name/initials, and any special characters (accents, umlauts, etc.). Please place first/given names before last/family names. We ask that you ensure they match verbatim; currently you have used initials only on the submission form, and full names on the submission form. Please be aware that correct spelling and formatting of author names in the article file is vital for accurate indexing in PubMed.**

Answer: Thank you, we have adjusted the names in the manuscript files to full names.

**2. Please ensure that all main figures are referenced in the manuscript as Fig 1, Fig 2, etc. (including capitalization), rather than Figure 1, Figure 2, etc. Please note, however, that the file names themselves must not include the space, i.e. Fig1.tif, Fig2.tif. Please ensure that all main figure files are clearly labeled as such in the File Inventory.**

Answer: Thank you, we have adjusted the manuscript accordingly.
